# Dietary Wood and Activated Charcoal Improved Ammonium Removal, Heavy Metals Detoxification, Growth Performance, Blood Biochemistry, Carcass Traits, and Histopathology of European Seabass

**DOI:** 10.1155/2023/8860652

**Published:** 2023-11-10

**Authors:** Ashraf I. G. Elhetawy, Mohamed M. Abdel-Rahim, Ahmed E. Sallam, Shimaa A. Shahin, Ayman M. A. Lotfy, Mohammed F. El Basuini

**Affiliations:** ^1^Aquaculture Division, National Institute of Oceanography and Fisheries NIOF, Cairo, Egypt; ^2^Animal and Fish Production Department, Faculty of Agriculture Saba-basha, Alexandria, P.O. Box 21531, Alexandria University, Egypt; ^3^Animal Production Department, Faculty of Agriculture, Tanta University, Tanta 31527, Egypt; ^4^Faculty of Desert Agriculture, King Salman International University, Tur Sinai, South Sinai, Egypt

## Abstract

A 120-day growth trial was completed to assess rearing water quality and fish performance in terms of growth, feed efficacy, digestive enzymes, immunity, and antioxidant activity of seabass fed an experimental diet (ED) supplemented with commercial wood charcoal (WC) and activated wood charcoal (AC). Three levels (0, 10, and 20 g) of WC and AC were administered, representing five treatments: control (CD) fish-fed ED without additives, (WC-1) fish-fed ED containing 10 g kg^−1^ WC, (WC-2) fish-fed ED containing 20 g kg^−1^ WC, (AC-1) fish-fed ED containing 10 g kg^−1^ AC, and (AC-2) fish-fed ED containing 20 g kg^−1^ AC. Three hundred fish (60.12 ± 0.20 g/fish) were stocked in 15 cement tanks (4.0 m × 2.0 m × 1.2 m, water volume 5 m^3^ each) at 20 fish/tank and a daily feed ration of 3% of body weight. Results revealed significant improvements with increased growth variables (final weight, weight gain, and specific growth rate), decreased FCR, and decreased ammonia levels and heavy metals (Cu, Cd, Fe, Mn, and Zn) content in rearing water, muscle, and liver with fish fed WC and AC supplemented diets. Furthermore, considerable improvements in digestive enzymes, immunity, and antioxidant activity, with enhanced kidneys, liver, intestines, gills, and spleen. Fish fed the WC-1 diet had a higher final weight (171.90 g), better FCR (1.25), and improved internal organs than the other groups.

## 1. Introduction

The diversity of aquaculture systems supports the role of aquaculture in providing a large proportion of animal protein of high nutritional value and quality [1], sustaining endangered species such as sturgeons and enabling farmers to produce fish outside their native habitat [2, 3]. Global aquaculture production was 122,600,000 tons in 2020, totaling 281.5 billion US$ [4]. Egypt's aquaculture ranks sixth globally, with an output capacity of 1,591,900 t, which is the largest in Africa, as it represents 67.62% of Africa's contribution to global aquaculture production, which amounted to 23,543 thousand tons in 2020 [4]. Aquaculture contributed to more than 79% of Egypt's total fish production in 2020, estimated at 1,588,966 t [5].

The global harvest of European seabass (*Dicentrarchus labrax*) has steadily increased from around 60,000 t in 2003 to 243,900 t in 2020, representing 2.9% of total global aquaculture production [4]. Egypt is the third-largest producer, with 34,477 t, after Turkey and Greece, whose combined production accounts for more than 69% of global production [5]. Seabass production is divided into 32,555 t from aquaculture, 258 t from the Mediterranean Sea, and 1,664 t from lakes [5]. After gilthead seabream (*Sparus aurata*) and meagre (*Argyrosomus regius*), seabass is Egypt's third most important aquacultured marine fish [5]. Seabass is farmed in Egypt using earthen ponds and cages, and a limited number of farmers use concrete ponds and flow-through systems along the Mediterranean's northern shore and northern lakes [6]. Seabass is an aquatic carnivorous that has been reared in captivity and fed artificial diets since the 1970s [7]. Thus, given the rising cost of feed ingredients and the global scarcity of fishmeal, it is critical to supplement diets with inexpensive additives that can improve performance while not impairing the aquatic environment.

Several studies have been conducted on grown fish using non-nutritive feed additives as detoxifying agents such as active charcoal to increase growth, improve health, and generate optimum environmental conditions by minimizing stresses [8, 9]. By absorbing pollutants and easing their elimination from the body, these feed additives can protect fish from the toxicity of heavy metals (HMs), resulting in better fish growth and immunity [6, 8]. One of the most useful non-nutritional feed additions is charcoal, and it is used in a process called chelation (a process in which a chemical or natural compound binds to a metal ion and holds it firmly) [10]. Charcoal is a crystalline form of carbon, typically resulting from the combustion of wood, bamboo, cellulose, coconut shells, or other carbonaceous byproducts of other industrial processes. It comprises 70%–90% crystalline carbon and many minerals, including sodium, copper, zinc, manganese, magnesium, calcium, potassium, and iron [8, 11]. It can absorb gases, particularly nitrogen and ammonia, stimulate intestinal function, and eliminate toxins and pollutants from animals' gastrointestinal tracts [12]. Several charcoal forms exist, including coconut shell charcoal, barbecue charcoal, activated bamboo charcoal (BC), activated charcoal (AC), and wood charcoal (WC) [13, 14].

AC is a fine black powder made by heating charcoal at a high temperature. The heating process to form AC increases the surface area by reducing the pore size resulting in a higher number of pores than other forms of charcoal. WC is a lightweight, solid black composite made by vigorously heating the woody parts of plants [13, 14]. Various types of AC have been utilized extensively as an effective detoxifying agents in the medical sciences, veterinary medicine, and aquatic medicine [8, 9, 15]. In addition, AC has been demonstrated to effectively eliminate mycotoxins, such as aflatoxins and pesticide residues [11]. Numerous studies have documented the use of AC, particularly as a feed additive in fish meals, with good effects on fish growth, health, and the aquatic environment [8–10]. The beneficial effects of incorporating AC into fish diets have been demonstrated using Nile tilapia *Oreochromis niloticus* [8, 16, 17], African catfish *Clarias gariepinus* [15], beluga sturgeon *Huso huso* [10], giant trevally *Caranx ignobilis* [18], and giant trevally juveniles [19]. Other forms of activated charcoal, such as BC, significantly increased the growth of tiger puffer fish *Takifugu rubripes* and olive flounder *Paralichthys olivaceus* [20, 21] and decreased the concentrations of nitrogen and ammonia in the farming water of striped catfish *Pangasiaodon sp*. [22]. For WC, the limited literature available on the incorporation in fish feed indicated its positive impact in improving the body composition of *P. olivaceus* [23], reducing the ecological load in the breeding water of red tilapia *Oreochromis mossambicus* × *Oreochromis niloticus* and gilthead seabream [24, 25].

The short- and long-term administration of charcoal to animals revealed no negative effects [26, 27]. On another hand, there may be some rarely occurring negative effects with long-term charcoal feeding, including: (1) changes in the species composition of microorganisms in the digestive system and (2) the possible adsorption of essential feed compounds and/or drugs, such as the immobilization of liposoluble feed ingredients, e.g., vitamin E or Carotenoids [27]. Although activated charcoal adsorbs strains of normal, healthy bacterial flora, their adsorption was significantly lower than that of the dangerous Gram-negative *Escherichia coli* [28]. Pathogenic microbes are more strongly attached to charcoal in the digestive tract than beneficial natural intestinal flora [28, 29]. Fujita et al. [30] conducted an in-depth study to investigate the effects of charcoal feeding on hen health and egg quality. When hens were fed 0.5% charcoal daily, the fat-soluble vitamins A and D3 did not show a statistically significant trend toward decreasing amounts in the egg yolk. However, the vitamin E content in the eggs decreased by around 40% when hens were fed 0.5% charcoal daily. Furthermore, changes in egg yolk color were associated with a decrease in carotenoids [31].

Many seabass farms grow these valuable marine fish in salt and/or brackish groundwater. However, seabass farmers who rely on these waterways believe that the water is contaminated with HM. The cost of purifying this water significantly impacts the breeder's earnings. Furthermore, no attempt has been made to investigate the impacts of charcoal as a dietary supplement on cultured marine aquatic organisms such as seabass. This work aims to assess the effects of adding AC and WC to seabass diets on growth performance, HM uptake, water quality, immunological parameters, blood biochemistry, and histological characteristics of fish grown in ground saltwater.

## 2. Materials and Methods

### 2.1. Animal Care Ethics

This investigation was operated at the El-Max Research Station of the National Institute of Oceanography and Fisheries (NIOF). All experimental procedures involving live fish were performed under Institutional Animal Care and Use supervision and were authorized by Alexandria University Animal Ethics Committee (IACUC approval 19/22/09/11/3/23).

### 2.2. Experimental Design and Diets

Five treatments were administrated using an isonitrogenous and isolipidic experimental diet (ED) formulated and supplemented with WC and AC at different concentrations (Table 1), as follows:

  (CD) control group where fish fed ED without any additives of WC and AC,
  (WC-1) where fish-fed ED containing 10 g kg^−1^ WC,
  (WC-2) where fish-fed ED containing 20 g kg^−1^ WC,
  (AC-1) where fish-fed ED containing 10 g kg^−1^ AC, and
  (AC-2) where fish-fed ED containing 20 g kg^−1^ AC.

The WC source (containing 85% carbon) was provided by First Logistic Egypt Co., Egypt, and AC was purchased from Jacobi Co., Sweden. The WC and AC tested levels referred to the findings of earlier studies [8, 10].

### 2.3. Fish and Rearing Management

Seabass juveniles were bought from the Kilo 21 Marine Fish Hatchery (GAFRD) and kept in 15 cement tanks (4.0 m × 2.0 m × 1.2 m, water volume 5 m^3^ each). Fish were adapted with the basal diet (CD) for 15 days. Before the initiation of the experiment, the fish were immediately weighed as a whole and in groups of 20 samples each to obtain information about the initial size and biomass in the tank. Three hundred healthy fish were selected and randomly divided into five groups of three replicates each, with an initial body weight of 60.12 ± 0.20 g and an initial length of 19 ± 0.4 cm. Twenty fish were stocked in each tank. For 4 months, fish were fed a trial diet at 3% of their body weight thrice daily, at 9:00, 12:00, and 15:00 hr. During the experiment, the daily water exchange value was 5%, and the fish were exposed to a natural light and dark cycle.

### 2.4. Water Quality Parameters

Water temperature, conductivity, pH, and dissolved oxygen (DO) were measured on-site using a multiparameter portable instrument (Lovibond model SensoDirect 150, Dortmund, Germany). Total NH_4_ was determined with the ammonia medium-range analyzer H196715 (HANNA Instruments, Romania) every 2 weeks at 17:00 hr (2 hr after the last meal). The content of HM in water was detected using an atomic absorption spectrophotometer (Agilent, Model AA280FS, USA). The samples were prepared and evaluated for zinc, copper, iron, manganese, and cadmium in the form described by APHA [32].

### 2.5. Analysis and Measurement

#### 2.5.1. Biometric Indices Assessment

After a 4-month growth trial, the total number and whole-body weight of fish in each tank were assessed to calculate growth performance parameters. At the end of the experiments, three fish per replicate tank were randomly chosen and sacrificed to measure the weights of the liver and viscera to calculate the hepatosomatic index (HSI) and viscerosomatic index (VSI).

#### 2.5.2. Proximate Chemical Analyses and HM Analyses

Proximate chemical analyses of whole-body fish composition were performed for moisture, crude protein, total lipids, and ash using 15 fish at the beginning of the trial and five fish per replicate tank at the end of the experiment, as well as the formulated diets, were analyzed according to AOAC [33]. HM concentrations (*µ*g/g dry weight) in the whole-fish body and liver (three fish per replicate tank) were measured by an atomic absorption spectrophotometer, as reported by Tüzen [34]. Water analysis of HM was performed according to Shkinev et al. [35].

#### 2.5.3. Blood Sampling and Biochemical Parameters Assessment

At the end of the growth trial, blood samples (three fish per replicate) were drawn from the caudal vertebral vein of an anaesthetized fish (100 mg/L MS222) using a 3 mL medical syringe and kept to clot at 4°C for 1–2 hr. Following centrifugation (3,000 r/min, 10 min, 4°C), the serum was separated and frozen at −20°C until use. All biochemical analyses of serum were completed using colorimetric techniques. The total protein (TP) and albumin (ALB) levels in serum were determined by Henry [36] and Doumas et al. [37] methods. Serum globulin was calculated by subtracting the albumin value from the total protein value of the same sample. The liver enzyme activity aspartate aminotransferase (AST), glutamic–pyruvic transaminase (ALT), and alkaline phosphatase (ALP) in serum were measured according to the method described by Reitman and Frankel [38]. The serum complement (C3) levels were assayed using C3 kits as described previously [39], and kidney function indicators (uric acid, blood urea nitrogen, and ammonia) were measured according to Whitehead et al. [40]. Serum glucose was detected by a semiautomatic biochemical analyzer (BAS-100 TS; Thomas Scientific, USA) according to Trinder [41]. Catalase (CAT, EC 1.11.1.6) enzyme was estimated according to the method of Aebi [42]. Using an automatic biochemical analyzer, total antioxidative capacity (TAC) activities were assessed (Hitachi 7600D, Hitachi, Tokyo, Japan) using the method of Koracevic et al. [43]. Superoxide dismutase (SOD, EC 1.15.1.1) was assessed according to the method of Shahin et al. [44]. Malondialdehyde (MDA) levels were determined according to Mihara and Uchiyama [45]. Glutathione peroxidase (GPx, EC 1.11.1.9) activity was detected using the method of Paglia and Valentine [46]. Activity of digestive enzymes (lipase and amylase) was measured according to methods described by Abdel-Tawwab et al. [47].

#### 2.5.4. Liver, Intestine, Spleen, and Gill Histological Processing

The liver, intestine, spleen, and gill of fish (three fish per replicate tank) were taken and submerged in 4% paraformaldehyde for 24 hr before being transferred to a series of ethanol concentrations to dehydrate the fixed samples in a gradient manner. Following dehydration, the tissues were rendered transparent with xylene before being set in an embedding machine, and sections of 3–5 *µ*m thickness were sliced with a microtome. The sections were stained with hematoxylin and eosin (H&E) and sealed with neutral resin [48]. The examination used an Olympus IX71 light microscope and a digital camera (C-4000 zoom).

### 2.6. Calculation Formula for Relevant Indicator

Growth performance and somatic indices were evaluated as follows:

1. Weight gain (WG, %) = 100 × (final body weight − initial body weight)/initial body weight.
2. Specific growth rate (SGR, %) = 100 × (ln(final body weight) − ln(initial body weight))/test days.
3. Feed intake (FI, g/fish/day) = feed consumption (g)/average biomass (g) × days.
4. Feed conversion ratio (FCR) = total feed consumption (g)/total weight gain (g).
5. Protein efficiency ratio (PER, %) = 100 × (total weight gain (g)/protein intake (g)).
6. Protein productive value (PPV, %) = 100 × (protein gain (g)/protein intake (g)).
7. Energy utilization (EU, %) = 100 energy gain (kcal)/energy intake (kcal).
8. Viscerosomatic index (VSI, %) = 100 × (viscera weight (g)/fish body weight (g)).
9. Hepatosomatic index (HSI, %) = 100 × (liver weight (g)/fish body weight (g)).
10. Intraperitoneal fat ratio (IPF, %) = 100 × (intraperitoneal fat weight (g)/fish body weight (g)).
11. Condition factor (CF, g/cm^3^) = final body weight (g)/final body length (cm)^3^.

### 2.7. Data Statistics and Analysis

The results were presented as mean ± standard error of the mean (SEM). All data were subjected to one-way analysis of variance (ANOVA) using SPSS Ver. 26.0 (SPSS Inc., Chicago, IL, USA). The Shapiro–Wilk and Levene tests tested data for normality and variance homogeneity. The statistical differences among treatments were confirmed with Tukey's multiple range test. The level of 0.05 was applied to state significant differences (*P* < 0.05).

## 3. Results

### 3.1. Influences on Water Quality

**Figure 1:**
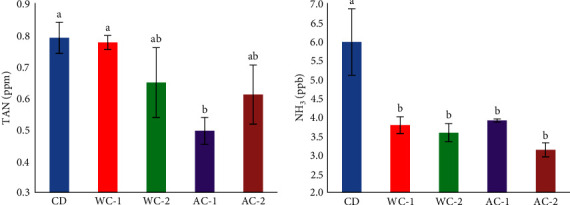
Total ammonia nitrogen (TAN, ppm) and unionized ammonia (NH_3_, ppb) in the rearing water of European seabass fed with different levels of commercial wood charcoal (WC) and activated charcoal (AC), for 120 days. Where CD = control group (experimental diet without any additives of WC or AC), WC-1 = experimental diet (containing 10 g WC kg^−1^ feed), WC-2 = experimental diet (containing 20 g WC kg^−1^ feed), AC-1 = experimental diet (containing 10 g AC kg^−1^ feed), and AC-2 = experimental diet (containing 20 g AC kg^−1^ feed).  ^*∗*^Values (mean ± SEM; *n* = 8 readings) represented with different superscript alphabets are significantly different (*P* < 0.05).

The addition of both WC and AC throughout the experiment had no significant effects on the physiochemical parameters, which ranged from 20.3 to 20.6°C, 7.2 to 7.5 mg/L of dissolved oxygen, 7.11 to 7.28 mg/L of pH, and 35.17 to 35.40 ppt of salinity (Table 2). However, the use of both WC and AC as a dietary supplement demonstrated a significant (*P* < 0.05) improvement as the concentrations of nonionized ammonia (NH_3_) were significantly lower in the WC and AC enriched groups than in the CD treatment. The four examined groups (WC-1, WC-2, AC-1, and AC-2) achieved the lowest values of NH_3_, with no significant changes among them. The lowest numerical value of total ammonia nitrogen (TAN) was recorded for the AC-1 group, with no statistically significant differences with the WC-2 and AC-2 groups (Figure 1). The level of NH_3_ was greater in the CD group.

### 3.2. Impacts on HM Detoxification

#### 3.2.1. HM Content in Rearing Water

**Figure 2:**
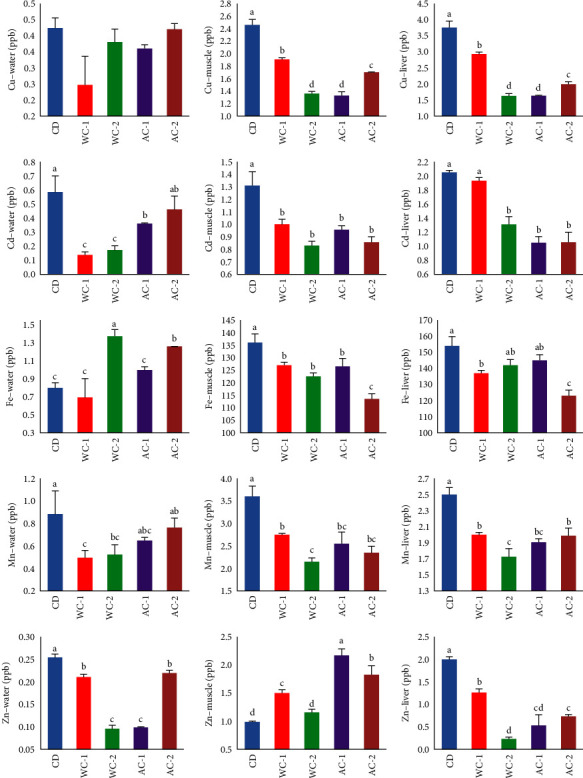
Heavy metal (Cu, Cd, Fe, Mn, and Zn) contents measured in rearing water, muscle, and liver of European seabass fed different levels of commercial wood charcoal (WC) and activated charcoal (AC), for 120 days. Where CD = control group (experimental diet without any additives of WC or AC), WC-1 = experimental diet (containing 10 g WC kg^−1^ feed), WC-2 = experimental diet (containing 20 g WC kg^−1^ feed), AC-1 = experimental diet (containing 10 g AC kg^−1^ feed), and AC-2 = experimental diet (containing 20 g AC kg^−1^ feed). Values (mean ± SEM, *n* = 3 readings) represented with different superscript alphabets are significantly different (*P* < 0.05).

The addition of WC and AC at various levels improved the absorption of HM (Cu, Cd, Fe, Mn, and Zn) from the rearing water, leading to a significant (*P* < 0.05) reduction in the concentration of these metals. Each level of AC and WC showed a different impact on the adsorption capacity of HMs, relying on the type of metal and the level of AC or WC, as exhibited in Figure 2. Compared to CD and the other groups examined, the WC-1 group showed the best results for detoxifying HM from the rearing water, with the lowest amounts of Cu, Cd, Fe, and Mn recorded. Except for the WC-1 group, Cu uptake was not significantly different between the groups tested. The WC-2 and AC-1 groups reported the lowest Zn values. Meanwhile, WC-1 and WC-2 reported the lowest Cd concentrations, while it was not significantly different between CD and AC-1. The Mn concentration was not significantly different between WC-1, WC-2, and AC-1 and between CD and AC-2. Also, Fe was not significantly different between CD and WC-1 and between CD and AC-2.

#### 3.2.2. HM Content in Seabass Muscle

The HM concentration detected in the muscle of seabass fed different levels of AC and WC is displayed in Figure 2. Except for Zn, whose concentration was lower in the CD group, supplementation of seabass diet with AC or WC increased HM removal from the fish muscle, which recorded lower levels in all groups compared to the CD. Higher removal of Cu was reported for the AC-1 (1.33 ppb) and WC-2 (1.36 ppb) groups, with no significant differences between them. At the same time, a lower level of Cu was recorded in the AC-2 group compared to the WC-1 group and CD, which had the highest Cu value. A significantly higher level of Cd (1.31 ppb) was observed in the CD group compared to the WC and AC enriched groups (WC-1, WC-2, AC-1, and AC-2) which had no significant change between them. The AC-2 group had the lowest Fe concentration (113.5 ppb). It varied significantly compared to the CD group, which had the highest level and compared to the other groups (WC-1, WC-2, and AC-1), which maintained the same level. Lower concentrations of Mn (2.15 ppb) and Zn (1.16 ppb) were reported for the WC-2 group, which did not differ significantly from AC-1 and AC-2 concerning Mn and CD regarding Zn. Except for Zn, the CD group had greater values for all HM muscle measurements than the AC and WC treatments.

#### 3.2.3. HM Content in Seabass Liver

HM content in the liver of seabass varied significantly (*P* < 0.05), as shown in Figure 2. The lowest significant values of Cu were recorded for WC-2 and AC-1 groups, with no significant differences between them. The WC-1 and AC-2 groups had significantly lower values than the CD group and significantly higher than the WC-2 and AC-1 groups. Moreover, lower values of Cd were reported for WC-2, AC-1, and AC-2, with no significant differences among them, while the CD and WC-1 groups shared a significantly higher Cd concentration. A significant decrease in Fe (123.0 ppb) was recorded in the AC-2 group, followed by the WC-1 group, while no statistically significant differences were recorded between the WC-2, AC-1, and CD groups. The lowest concentrations of Mn (1.68 ppb) and Zn (0.23 ppb) favored the WC-2 group, while the AC-1 group shared a significantly lower concentration of Zn with the WC-2 group. Compared to the AC and WC treatments, the CD group had statically higher values of Cu and Zn. In comparison, it had numerically higher values of Cd with no significant differences with WC-1, Fe with no significant differences with WC-2 and AC-1, and Mn with no significant differences with AC-1.

### 3.3. Growth Performance, Feed Utilization, and Biometric Indices of Seabass

The mean ANOVA analysis revealed that the level of WC or AC had a significant (*P* < 0.05) impact on the growth performances and feed utilization of seabass fed a diet supplemented with varying levels over a 4-month growth trial (Table 3). Fish fed WC or AC-supplemented diets performed significantly better than the CD group. The inclusion of WC at 10 g kg^−1^ in the diet of seabass increased growth and improved FCR much more than fish in the CD group that did not receive WC or AC, as well as other tested fish groups (WC-2, AC-1, and AC-2). When compared to the CD and other studied groups, adding 10 g kg^−1^ WC to the diet in the WC-1 group resulted in the largest increases in FW (171.90 g), WG (111.82 g), and SGR (0.876). Furthermore, except for the WC-1 group, the AC-1 group showed a significant increase in FW (159.67 g), WG (99.73 g), and SGR (0.817) as compared to the CD and other examined groups. The AC-2 group shared the lowest FW, WG, and SGR findings with the CD group, with no significant differences. The addition of 10 g kg^−1^ WC and AC to the diet of seabass (WC-1 and AC-1) significantly increased WG% (186.3% and 166.4%, respectively) compared to 156.3%, 161.2%, and 153.2% in the CD, WC-2, and AC-2 groups. All groups examined had a 100% survival rate for fish.

Regarding the feed utilization of seabass, adding WC significantly improved FCR, achieving the best value (1.25) with fish fed on a WC-1 diet. Moreover, fish fed the WC-2 diet had a better FCR (1.44) than fish fed the CD and AC-2 diets. Furthermore, by including AC in the diet, the FCR improved in the AC-1 group, with fish fed 10 g kg^−1^ AC having a significantly higher value (1.49) than the CD and AC-2 groups. A similar pattern of findings was shown for PER and PPV as for FCR. The fish in the WC-1 group had the highest PER value (1.76), followed by those in the WC-2 and AC-1 groups, with no significant differences. Table 3 shows that the WC-1 fish group had higher PPV, energy gain, EU, and CF values than the other test groups (CD, WC-2, AC-1, and AC-2). Regarding FCR, PPV, PER, energy gain, and EU, there was no significant difference between the WC-2 and AC-1 groups. However, the CF differed significantly in favor of the WC-1 group. HSI values of supplemented groups with charcoal were superior to that of the CD group. The highest VSI value was reported for fish fed the AC-2 group with no significant differences with other groups except WC-2. A higher IPF value was reported for the AC-2 fish group, which differed significantly from the other tested groups, except for CD.

### 3.4. Whole-Body Chemical Composition

The whole-body chemical composition of the seabass carcass is presented in Table 4. Statistical analysis revealed that adding WC or AC had a significant effect (*P* < 0.05) on the main components of seabass carcass. Fish fed both WC-2 and AC-1 diets showed a significant increase in protein content than fish in CD and the other tested groups. Moreover, there were no significant variations regarding protein content in fish fed the CD diet and those fed the WC-1 and AC-2 diets. The highest dry matter content was reported for fish in the AC-2 group, while no significant differences were recorded for fish fed on CD, WC-1, and WC-2 diets and between fish fed the CD and AC-2 diets. Fish fed the AC-2 diet showed higher content of lipids, while no statistical differences were stated between fish fed the CD, WC-2, and AC-1 diets or between WC-1 and WC-2 diets. Higher carcass energy was observed in fish fed the AC-2 diet, with no statistical variation in the WC-1 group. Moreover, adding AC and WC at different doses did not significantly change ash content, with CD treatment having the highest value.

### 3.5. Effects on Blood Hematological and Biochemical Parameters

The use of AC and WC as feed additives significantly (*P* < 0.05) improved the kidney and liver function as well as blood biochemical parameters of *D. labrax*. Regarding kidney function indicators, significant decreases in urea, uric acid, and ammonia levels were recorded for fish-fed AC or WC-supplemented diets compared with the CD group (Table 5). The lowest level of urea (13.0 mmol/L) was recorded for fish raised in the AC-2 group. Lower values of uric acid (2.20 and 2.38 mg/dL) were observed in both AC-2 and WC-2, respectively, with no significant differences. In contrast, all tested groups (WC-1, WC-2, AC-2, and AC-2) shared lower ammonia values with no significant differences. The highest levels of urea (21.0 mmol/L), uric acid (3.41 mg/dL), and ammonia (34.50 mmol/L) were reported for fish-fed CD diet. Regarding liver enzymes (ALP, ALT, and AST), fish fed the tested diets (WC-1, WC-2, AC-1, and AC-2) exhibited lower values compared to fish fed the CD diet (Table 5). Lower values of ALP (64.0 U/L), ALT (24.5 U/L), and AST (49.0 U/L) were reported for fish grown in the WC-2 group compared with other tested groups. Moreover, fish fed with the WC-1 diet shared a lower ALP value with fish fed the WC-2 diet, while fish under the WC-2 and AC-2 diets shared lower values of AST, with no significant differences. Fish fed the CD diet exhibited higher values (*P* < 0.05) of ALP (84.5 U/L), ALT (49.5 U/L), and AST (78.5 U/L). The composition of the seabass whole-body carcass is presented in Table 4.

Furthermore, including AC and WC in the diets significantly (*P* < 0.05) affected the activity of digestive enzymes and antioxidant enzymes in the fish body (Table 6). The WC-1 group had the highest activity of lipase (25.33) and amylase (52.00), but the activity of both enzymes improved significantly in the other evaluated groups compared to the CD group. A significant decrease (*P* < 0.05) in serum antioxidant enzymes TAC, SOD, and CAT and a significant increase (*P* < 0.05) in MAD and GPx activity in fish-fed AC and WC-supplemented diets compared to fish of CD diet. Additionally, compared to fish fed the CD diet, the immunological serum indices improved significantly in those fed AC and WC diets (Table 7). Total protein (185.0 mg/dL) was higher in the AC-2 group, albumin (1.67 and 1.655 mg/dL) in the AC-2 and WC-1 groups, respectively, and C3 (12.4 and 11.75 mg/dL) were higher in the WC-1 and AC-2 treatments. Furthermore, all tested groups revealed a significant rise in globulin levels compared to the CD group. In contrast, including WC and AC had a detrimental effect on the hemoglobin coefficient, with a substantial rise (*P* < 0.05) for fish fed the CD diet.

### 3.6. Influences on the Histological Features of the Intestine, Gills, Liver, and Spleen

**Figure 3:**
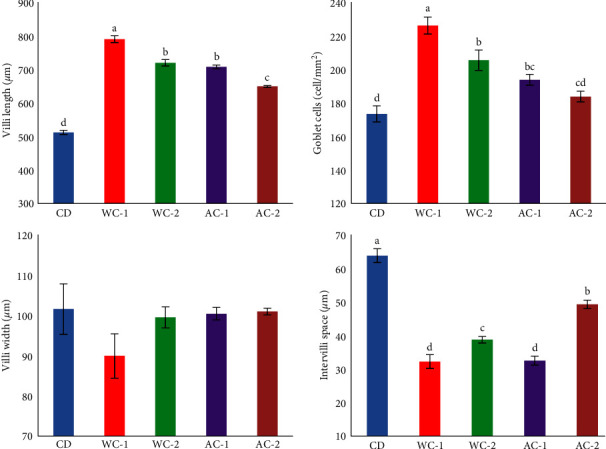
Histological data of middle intestine (villi length, villi width, intervilli space, and goblet cells) of European seabass fed different levels of commercial wood charcoal (WC) and activated charcoal (AC), for 120 days. Where CD = control group (experimental diet without any additives of WC or AC), WC-1 = experimental diet (containing 10 g WC kg^−1^ feed), WC-2 = experimental diet (containing 20 g WC kg^−1^ feed), AC-1 = experimental diet (containing 10 g AC kg^−1^ feed), and AC-2 = experimental diet (containing 20 g AC kg^−1^ feed).  ^*∗*^Values (mean ± SEM, *n* = 3 readings) represented with different superscript alphabets are significantly different (*P* < 0.05).

**Figure 4:**
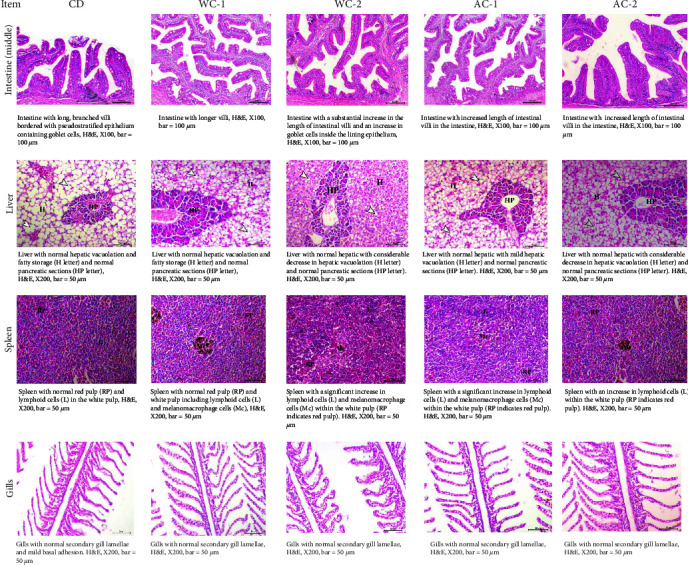
Histology of middle intestine, liver, spleen, and gills of sea bass fed different levels of commercial wood charcoal (WC) and activated charcoal (AC). Where CD = control group (experimental diet without any additives of WC or AC), WC-1 = experimental diet (containing 10 g WC kg^−1^ feed), WC-2 = experimental diet (containing 20 g WC kg^−1^ feed), AC-1 = experimental diet (containing 10 g AC kg^−1^ feed), and AC-2 = experimental diet (containing 20 g AC kg^−1^ feed).

Figures 3 and 4 show the histological results of the middle intestines, liver, gills, and spleen. Seabass fed on diets supplemented with WC and AC showed a significant (*P* < 0.05) increase in villi length, intervilli space, and goblet cells in the middle intestine (Figure 3). The best results favored treatments WC-1, WC-2, AC-1, and AC-2, while CD exhibited the worse results. The treatments WC-1, WC-2, AC-1, and AC-2 had the best results for the middle intestine, whereas CD produced the lowest results. The livers of fish from group CD exhibited normal hepatic vacuolation with fatty storage (H letter) and normal pancreatic portions (HP letter, Figure 4). In contrast, fish livers from groups fed WC and AC-supplemented diets exhibited normal hepatic vacuolation with a marked decrease and normal pancreatic portions (Figure 4). The spleen of fish in group CD had normal red pulp and white pulp composed of lymphoid cells, whereas the spleens of fish in groups given WC and AC-supplemented diets had a marked increase in the number of lymphoid cells and melanomacrophage cells inside the white pulp (Figure 4). The gills in all groups showed normal secondary gill lamellae, while the CD group showed mild basal adhesion (Figure 4).

## 4. Discussion

The growth and health of cultivated fish are directly proportional to the quality of their diets and surrounding environment [49], as well as the system utilized in farming [50]. In the present study, NH_3_ concentrations were considerably affected by WC and AC supplementation and decreased significantly in favor of the tested groups (WC-1, WC-2, AC-1, and AC-2) compared to the CD group. The decreased NH_3_ concentrations in both WC- and AC-enriched groups may be attributable to the efficiency of charcoal in ammonia absorption, although the leverage of uptake is dependent on the dose of WC or AC and the quantity of gases in the gastrointestinal tract [8, 51]. The findings of lower NH_3_ concentrations in the WC and AC-fed fish groups are consistent with those in Thu et al.'s [20, 21] study, who found that dietary BC lowered ammonia-N levels in puffer fish and *P. olivaceus*, respectively, as compared to the CD group. Similarly, Michael et al. [24] found a significant reduction in ammonia concentrations in the rearing water of red tilapias fed diets supplemented with 1%, 2%, 3%, and 4% WC for 60 days.

Moreover, Michael and Helal [25] reported the positive effect of including WC in the diet of sea bream at 20 and 40 g/kg at different stocking densities, noting a gradual decrease in ammonia levels in the growing water of fish groups stocked at 40 and 60 fish m^−3^, compared to the CD treatment. In this study, AC and WC-supplemented diets positively decreased ammonia levels, with no significant differences between the higher levels (WC-2 and AC-2) and lower levels (WC-1, AC-1). This differs slightly from the conclusion that greater dietary charcoal in fish feed causes a decrease in TAN excretion [21, 24, 52]. The differences between the recommended levels of WC and AC may be attributable to variances in culture practices, water quality standards, fish species, and the quality of dietary protein sources.

Management practices, rearing techniques, and the environment influence fish growth during the growth period [50, 52–55]. Dietary supplementation of the seabass diet with varying levels of WC and AC improved fish growth and feed utilization indices significantly. However, HM contamination in the rearing water (Figure 2) is the leading cause of the inability to triple weight, which is the gold standard for growth trials, as previously documented [56]. In our trial with HM contamination, using charcoal supplements achieved final weights of 171.90 and 159.67 g equaling 2.86 and 2.66 times the initial weight in WC-1 and AC-1, respectively. In other words, fish fed the WC-1 and AC-1 diets increased their FW by more than 19.6% and 6.7%, respectively, compared to those fed the CD diet. The current study found that 10 g kg^−1^ is the appropriate level of WC inclusion in the diet of seabass and that increasing the levels of WC and AC inclusion from 10 to 20 g kg^−1^ did not increase growth and FCR parameters. Following the findings of Yoo et al. [23], the best level of a mixture of food charcoal and wood vinegar (CV82; 80% charcoal and 20% vinegar) for optimal growth of Japanese flounder was 0.5%–1.0% of the diet. Similarly, Thu et al. [21] indicated that the best dietary charcoal level for juvenile Japanese flounder was 0.5%, whereas the optimal dietary AC level was recommended to be 7.0 g/kg diet for Nile tilapia [8]. Close to the levels mentioned above, Samadaii and Bahrekazemi [10] found that the addition of 1.5% AC kg^−1^ to the diet significantly improved the growth and nutritional parameters of big sturgeon, while the addition of 2% AC was determined to be optimal for promoting growth and intestinal morphology in Nile tilapia [17]. Aside from these ranges, it was determined that 4% BC is the best level to include in tiger puffer fish diets, according to Thu et al. [20], and adding 3% WC to the feed of juvenile red tilapia was ideal for fish growth and feed utilization [24]. Growth performances of Nile tilapia (380–460 g) fed on diets containing 0, 10, 20, and 30 g AC/kg diet were not significantly different from each other, as revealed by, Boonanuntanasarn et al. [16], which contradicts earlier findings.

The mechanism of charcoal in fish digestion is unknown. However, the favorable effects of charcoal on the digestion and absorption of nutrients in fish can explain the improved findings observed here, particularly the improvements in the fish gut as evidenced by increased villi length, villi width, intervilli space, and goblet cells in fish-fed AC and WC-supplemented diets. The adsorption potential of WC to remove harmful gases and toxins from the intestine, enhancing diet digestion and metabolism, may contribute to enhancing growth and feed intake [16, 24]. In addition, dietary charcoal supplementation can improve the absorption function of intestinal villi and intestinal epithelial cells, hence enhancing feed utilization [12], as well as enhancing the height of intestinal villi in comparison to the CD group [16, 17]. There was a correlation between villi height and growth performance in Nile tilapia, with longer villi resulting in a greater surface area capable of improved nutrient absorption [12]. In the current study, HSI increased significantly with increasing WC and AC levels up to 20 g/kg. The higher results in this study with increasing WC and AC dosage could be attributed to increased liver metabolic activity. Sadekarpawar and Parikh [57] explained this conclusion due to the high binding capabilities increasing digesting viscosity and metabolic rate.

The results showed that both WC and AC improved the performance and chelation of seabass rearing waters, whereas WC-2 and AC-1 did not have significant differences concerning FCR and PPV. However, adding WC at the level of 10 g/kg diet showed greater superiority than AC, resulting in a considerable improvement of FCR, PPV, and PER. This could be explained by AC being produced when regular charcoal is burned to a very high temperature. As a result, the elements and compounds (moisture and beneficial volatile matter) bound to the carbon atoms are eliminated, and all the carbon binding sites are “free” to bind with incoming molecules and atoms [58]. This makes AC significantly more porous, substantially expanding its surface area, and making it far more effective at filtering material and as an adsorbent than charcoal. Therefore, AC eliminates undesired elements from water or feed, especially when their concentration is large.

Nonetheless, if the concentration of undesirable elements is moderate or low, the high adsorption capability of AC may pose challenges for cultivated organisms. In this experiment, the enhanced performance of seabass fed a 10 g/kg supplemented food compared to those fed a 20 g/kg supplemented diet was noticeable. The findings of this study concerning the difference in effect are consistent with previous research [59]. Additionally, variations in the source and nature of the charcoal, rearing conditions, the quantity of HM in the water, fish species, fish weights and ages, and feeding habits could all contribute to differences in the optimal level of charcoal among experiments. Additionally, charcoal's adsorption capacity depends on the contact time with the adsorbing compounds [60]. Furthermore, it was stated that the potential quantity of toxins in the gastrointestinal tract influences the efficacy of AC absorption and the dosage administered [51]. Also, Thu et al. [20, 21] reported that slow-eater fish differ from active-eater fish regarding dietary charcoal required to maintain optimal growth.

Previous research showed that fish accumulated more HM when its levels were higher in their environment or diet, especially in their gills, liver, and muscles [61]. Additionally, fish can accumulate HM several times greater than those in culture water [8, 62]. In the present experiment, even though the fish were grown in the same water source (well groundwater), Cu, Cd, Fe, Mn, and Zn residues in the muscle and liver of fish fed the WC-1, WC-2, AC-1, and AC-2 diets, as well as the culture water, were significantly reduced in comparison to those fed the CD diet. In addition, diets supplemented with 10 and 20 g of WC were more effective at removing HM from rearing water, fish muscle, and liver than diets supplemented with 10 and 20 g of AC. With the exception of a decreased Fe content in muscle and liver with the AC-2 supplemented diet, the WC supplemented diets had the lowest amounts of detected HM. Due to the lack of historical research comparing the efficacy of WC and AC in decontaminating fish bodies and culture water, it is difficult to compare the current results with those of the previous works. However, identical results were reported for Nile tilapia, indicating that increasing the amount of charcoal reduced HM accumulation in the fish body [8]. In addition, beluga fed a meal supplemented with 20 g/kg AC produced the greatest results in metal chelation, comparable to those produced by a diet supplemented with 15 g/kg AC [10].

The results of seabass carcass analysis showed that fish-fed diets supplemented with AC 10 g/kg and WC 20 g/kg had improved crude protein content than fish-fed WC 10 g/kg, AC 20 g/kg, and the CD diet. Also, adding AC at a level of 20 g/kg to the diet significantly raised the total lipid content in fish carcass than the CD and other groups. This result concurs with Samadaii and Bahrekazemi [10] findings, which showed that the composition of beluga carcasses with increasing charcoal content—up to 15 g/kg diet—was characterized by increased levels of protein and fat contents and lower moisture contents. For Nile tilapia, the same effects of enhanced protein content by increasing the dietary charcoal ratio were noted [8, 16], red tilapia [24], and Japanese flounder [21]. The authors attributed the increase in protein content to the positive effects of dietary charcoal on the digestion and absorption of nutrients, which led to a significant increase in crude protein content compared to the CD. Differences in the protein content of fish-fed AC-1 and WC-2 compared with those fed the WC-1 and AC-2 diets may be explained by the fact that AC is more active than WC, and increasing AC levels in the diet may negatively affect the digestion and absorption of protein. This varies from species to species of fish and depends on the dose provided of AC and WC. In contrast to what we found here, previous studies found that feeding charcoal did not have much effect on moisture, total lipid, and ash [16, 21, 24]. The decrease in moisture content is likely attributable to the increase in other carcass components, such as protein, lipid, and ash, whereas changes in the protein and lipid contents of the fish body are attributable to alterations in their average synthesis or degradation in the body [8].

In aquaculture research, blood quality measurements represent biochemical changes occurring in fish, revealing their metabolic and physiological status in general. Therefore, blood biochemistry measures are often utilized as diagnostic tools in biomonitoring, allowing for the detection of pathophysiological changes attributed to nutrition [16, 63]. The findings of the present study demonstrated a significant decrease in the kidney indicators, particularly (uric acid, urea, and ammonia) and liver enzymes (ALT, AST, and ALP), in treatments, fed WC and AC, particularly treatments WC-1, WC-2, and AC-1. This result is consistent with previous studies [8, 10, 61]. According to Abdel-Tawwab et al. [8], uric acid and creatinine are traditional diagnostic indices for kidney function and renal structural integrity. Thus, the higher quantities of uric acid and creatinine in fish are symptomatic of liver and renal disease, which leads to the leakage of these components from the respective organs into the bloodstream. Also, it is well-documented that ALT and AST are widely employed in diagnosing pollutant-induced damage to liver tissues [64]. According to Samadaii and Bahrekazemi [10], adding AC, particularly in treatments containing 15 and 20 g/kg feed of AC, can modify the hematological consequences if rearing water has greater concentrations of HM. Digestive enzymes are essential for nutrient absorption and feed utilization. The current study found a significant increase in the content of digestive enzymes (amylase and lipase) in fish given AC and WC diets. This enhancement could be attributed to charcoal's ability to remove harmful gases and toxins from the gut [16, 24], as well as improving the absorption function of intestinal villi and intestinal epithelial cells [12], resulting in increased metabolic activity of digestive enzymes. Both tilapia and sea beam showed similar increases in amylase and lipase levels [65].

The enzymes SOD, TAC, GPx, and CAT are the major antioxidant agents in the body. These enzymes may scavenge unwanted O_2^−^_ and H_2_O_2_, and ROOH produced by free radicals [8, 66]. In this study, considerable differences were noticed plasma TAC, SOD, and CAT values which increased significantly (*P* < 0.05) in the fish-fed CD diet compared to those fed WC and AC diets. Still, MAD and GPx activities were significantly higher in fish-fed WC or AC-supplemented diets than in fish-fed CD diet. Moreover, increasing the AC level from 10 to 20 g/kg increased MAD and GPx activities, while increasing WC from 10 to 20 g/kg increased GPx activity. These findings propose that the inclusion of AC and WC reduced the effects of HM on blood biochemistry and antioxidant activity in fish-fed supplemented diets resulting in decreased activity. In this regard, it was stated that alteration in the activities of antioxidant enzymes and the induction of lipid peroxidation reverses the effect of HM stress on the defense system of African catfish collected from the Ogun River, Nigeria [67]. In CD fish, the increased activity of antioxidant enzymes may echo HM-induced oxidative stress, as the bioaccumulation of HM in the fish body may lead to the formation of superoxide anions, which stimulate SOD to convert the superoxide radical into H_2_O_2_ [67]. The presence of HM may cause changes in the activities of MDA and antioxidant enzymes. Because the SOD-CAT system is regarded as the first line of defense against oxidative stress, an increase in SOD and CAT activity is typically observed in the presence of environmental contaminants [68]. These findings are consistent with those reported by Abdel-Tawwab et al. [8], who found that exposing Nile tilapia to environmental stress of HMs increased SOD, CAT, and GPX activities in fish fed a CD diet compared to those fed an AC-supplemented diet, and with those reported by Shahin et al. [44] for seabass larvae given silymarin (*Silybum marianum*) supplemented diets. The authors attributed the decreased activity of antioxidant enzymes in AC-fed fish to the key role played by AC in alleviating HM-induced oxidative stress.

Furthermore, GPx activity in fish-fed WC or AC-enriched diets was higher than that of the CD fish; these outcomes hypothesize that dietary WC or AC supplementation may promote fish health by scavenging free radicals and enhancing immune defense functions. These results are in line with Abdel-Tawwab et al. [8], who stated that prior to exposure of Nile tilapia to HM environmental stress, no significant differences were reported in MDA, SOD, and CAT levels between fish-fed AC enriched diet and that fed the CD diet. Still, GPx activity was significantly higher in fish fed a 20 g AC/kg diet than the CD fish. Moreover, Wang et al. [69] indicated that pigs fed diets containing different levels of bamboo vinegar showed no significant differences in serum MDA, SOD, and CAT values among different groups, but GPx activity was significantly higher at 0.2% or 0.4% bamboo vinegar levels.

In this study, including both WC and AC in the diet improved immunogenicity indices in seabass serum. This improvement is shown in higher levels of serum total protein, alkaline phosphate, albumin, and complement 3. Serum total protein level is a broad baseline clinical measure of health, stress, humoral defense system, and aquatic organism well-being [70]. Furthermore, blood proteins are required for fish to maintain blood pH and osmotic pressure [71, 72]. The increase in total protein level in fish-fed AC and WC-supplemented diets can be attributed to enhancements in the fish's digestive tract, which leads to an increase in synthesis and absorption capacities, as compared to the fish-fed CD diet, which had more pollutants in the water, which increased protein loss through blood dilution [73]. These findings were consistent with previous research [8, 10, 23]. Previous studies discovered that giving AC to fish improved their health and boosted their protein and lipid metabolism [8, 23]. No change in glucose levels among fish in all groups is consistent with previous studies [10].

Fish growth, disease resistance, stress tolerance, and feed efficiency are all affected by how well their internal organs develop, particularly their digestive tract, liver, gills, and spleen [49]. The current study found that adding AC and/or WC to seabass diets had a significant positive effect on the digestive system, liver, spleen, and gills. This is consistent with several previous studies [9, 24, 74]. According to Mabe et al. [9], adding BC to the feed of juvenile *Cyprinus carpio* has been found to improve the fish's digestive health and overall health. The previous authors discovered that at 4% supplementation, improvements in intestinal villus length and goblet cell counts were observed. Adding AC and/or WC to fish diets improves their health and the development of their internal organs. This is mostly due to the fact that AC aids in the adsorption of HMs [8], ammonia [24] by fish, and feedstuff toxicities, such as aflatoxin [75]. Therefore, the adsorption process improves growth performance, nutritional utilization, and the prevention of environmental and nutritional disorders. Also, charcoal functions as an adsorbent of different poisons, gases, and medications due to its vast surface area, which provides numerous bonding sites for the adsorption of many toxins and harmful gases [76]. However, Quaiyum et al. [52] explained that better nutrient utilization by using AC-supplemented feed is attributed to the growth-promoting microbes present along the gastrointestinal tract of animals. So, the basic function of charcoal is to affect the functions of digestion and absorption of nutrients positively. Few studies link the effect of adding AC and/or WC to the development of internal organs in aquatic organisms; therefore, more research is required in this area.

## 5. Conclusion

Non-nutritive feed additives are used in fish farming to achieve various aims, including increasing growth rates, boosting immunity, resolving concerns with feed raw material quality, and offering functional enhancers, such as resistance to environmental stress. This study found that adding 1%–2% WC or AC to a seabass diet has many benefits, including reduced ammonia excretion, improved growth rate and nutrient utilization, reduced content of HM in the fish's water, liver, and muscles, and significantly improved fish health, as evidenced by improvements in biochemical blood indicators and internal organs (gills—middle intestine—liver—spleen). Based on the superior increases in fish performance and the low cost compared to AC, WC inclusion at the level of 10 g/kg diet should be considered in the diet of sea bass. More research is needed to establish an optimal WC supplement level and test its applicability to various marine species.

## Figures and Tables

**Table 1:** Formulation and approximate chemical analysis of the experimental diets.

Items	Experimental diets				
	Control (CD)	Wood charcoal		Activated charcoal	
		(WC-1)	(WC-2)	(AC-1)	(AC-2)
Ingredients (%)					
Fish meal	20	20	20	20	20
Soybean meal	22	22	22	22	22
Corn gluten meal	18.2	18.2	18.2	18.2	18.2
Wheat flour	28.35	27.35	26.35	27.35	26.35
Fish oil	5	5	5	5	5
Vegetable oil	5	5	5	5	5
Binder	1	1	1	1	
Vit. and min premix^a^	0.3	0.3	0.3	0.3	0.3
Probiotic	0.1	0.1	0.1	0.1	0.1
Vitamin C	0.05	0.05	0.05	0.05	0.05
Wood charcoal (WC)	0	1	2	0	0
Activated carbon (AC)	0	0	0	1	2
Proximate composition^b^					
Moisture (%)	7.28 ± 0.09	7.27 ± 0.04	7.31 ± 0.11	7.29 ± 0.06	7.34 ± 0.04
Protein (%)	45.6 ± 0.71	45.15 ± 0.35	44.71 ± 0.88	45.14 ± 0.54	44.69 ± 0.22
Lipid (%)	16.5 ± 0.32	16.35 ± 0.11	16.19 ± 0.27	16.34 ± 0.10	16.17 ± 0.16
Ash (%)	14.42 ± 0.03	14.42 ± 0.04	14.42 ± 0.02	14.42 ± 0.01	14.42 ± 0.04
Fibers (%)	3.42 ± 0.08	3.51 ± 0.10	3.54 ± 0.06	3.50 ± 0.08	3.55 ± 0.10
NFE^c^	20.06	20.57	21.14	20.6	21.17
Gross energy, Kcal/1 kg DM^d^	4953.9	4935.3	4918.8	4935.1	4917.1
Gross energy, MJ/kg^e^	20.74	20.66	20.60	20.66	20.59

Treatments = control diet CD (0 g WC or AC kg^−1^ feed), WC-1 (10 g WC kg^−1^ feed), WC-2 (20 g WC kg^−1^ feed), AC-1 (10 g AC kg^−1^ feed), and AC-2 (20 g AC kg^−1^ feed). ^a^Vitamin and mineral premix: premix : vitamin A (3,300 IU), vitamin D3 (410 IU), vitamin B1 (133 mg), vitamin E (150 mg), vitamin B2 (580 mg), vitamin B6 (410 mg), vitamin B12 (50 mg), biotin (9330 mg), choline chloride (4,000 mg), vitamin C (2,660 mg), inositol (330 mg), para-aminobenzoic acid (9,330 mg),niacin (26.60 mg), pantothenic acid (2,000 mg), manganese (325 mg), iron (200 mg), copper (25 mg), iodine, cobalt (5 mg). ^b^Chemical analysis of three replicates. ^c^NFE is nitrogen free extract, NFE% = 100 − (moisture % + CF (fibers) % + CP (protein) % + EE (lipids) % + ash%) and DM is dry matter. ^d^gross energy, Kcal/1 kg was calculated using a value of 5.64 Kcal/g proteins, 9.44 Kcal/g fat, and 4.11 Kcal/g carbohydrates (NFE). ^e^1 kcal = 0.0041868 MJ.

**Table 2:** Water quality parameters in experimental tanks stocked with European seabass fed experimental diet supplemented with different levels of wood charcoal (WC) and activated charcoal (AC), for 120 days.

Parameters	Control (CD)	Wood charcoal		Activated charcoal	
		(WC-1)	(WC-2)	(AC-1)	(AC-2)
Temperature (T°C)	20.30 ± 0.57	20.35 ± 0.61	20.32 ± 0.41	20.55 ± 0.63	20.6 ± 0.51
pH	7.28 ± 0.11	7.11 ± 0.00	7.20 ± 0.12	7.44 ± 0.10	7.15 ± 0.06
DO (ppm)	7.21 ± 0.18	7.35 ± 0.14	7.20 ± 0.12	7.45 ± 0.18	7.50 ± 0.29
Salinity (g/L)	35.33 ± 0.12	35.17 ± 0.17	35.36 ± 0.02	35.40 ± 0.21	35.29 ± 0.44
TAN (ppm)^1^	0.790 ± 0.05^a^	0.775 ± 0.02^a^	0.648 ± 0.11^ab^	0.495 ± 0.04^b^	0.610 ± 0.09^ab^
NH_3_ (ppb)^2^	5.97 ± 0.88^a^	3.77 ± 0.22^b^	3.55 ± 0.25^b^	3.90 ± 0.04^b^	3.13 ± 0.18^b^

^1^TAN (ppm) = total ammonia nitrogen. ^2^NH_3_ (ppb) = nonionized ammonia. ^*∗*^Values are means ± SEM. *N* = 16 readings for T°C, pH, DO, and salinity. *N* = 8 readings for TAN and NH_3_. Values in the same row with a different superscript alphabets are significantly different (*P* < 0.05). Treatments = control diet CD (0 g WC or AC kg^−1^ feed), WC-1 (10 g WC kg^−1^ feed), WC-2 (20 g WC kg^−1^ feed), AC-1 (10 g AC kg^−1^ feed), and AC-2 (20 g AC kg^−1^ feed).

**Table 3:** Growth performance, feed utilization, and biometric parameters of European seabass fed an experimental diet supplemented with different levels of wood charcoal (WC) and activated charcoal (AC), for 120 days.

Parameter	Control (CD)	Wood charcoal		Activated charcoal	
		(WC-1)	(WC-2)	(AC-1)	(AC-2)
Growth, survival, and condition factor parameters					
Initial weight (g/fish)	59.75 ± 0.29	60.08 ± 0.65	60.18 ± 0.58	59.93 ± 0.52	60.27 ± 0.13
Final weight (g/fish)	153.18 ± 0.62^c^	171.90 ± 1.07^a^	157.87 ± 0.92^b^	159.67 ± 0.51^b^	152.60 ± 0.32^c^
Gain (g/fish)^1^	93.43 ± 0.58^d^	111.82 ± 1.10^a^	97.28 ± 0.60^c^	99.73 ± 0.94^b^	92.33 ± 0.39^d^
ADG (g/fish/day)^2^	0.779 ± 0.005^d^	0.932 ± 0.009^a^	0.811 ± 0.005^c^	0.831 ± 0.008^b^	0.769 ± 0.003^d^
SGR (%/fish/day)^3^	0.785 ± 0.005^cd^	0.876 ± 0.009^a^	0.798 ± 0.006^bc^	0.817 ± 0.009^b^	0.774 ± 0.003^d^
Survival (%)	100.00	100.00	100.00	100.00	100.00
Condition factor^4^	0.940 ± 0.003^b^	1.046 ± 0.008^a^	0.947 ± 0.011^b^	0.963 ± 0.051^b^	0.966 ± 0.017^b^
Feed utilization parameters					
FI (g/fish^−1^)^5^	166.306 ± 0.12 ^a^	139.775 ± 10.32 ^d^	140.084 ± 03.15^d^	148.598 ± 11.06 ^c^	153.268 ± 05.23^b^
FCR^6^	1.78 ± 0.02^a^	1.25 ± 0.04^d^	1.44 ± 0.02^c^	1.49 ± 0.01^c^	1.66 ± 0.02^b^
PER (g)^7^	1.23 ± 0.02^d^	1.76 ± 0.05^a^	1.52 ± 0.03^b^	1.47 ± 0.01^b^	1.32 ± 0.01^c^
PPV (%)^8^	20.43 ± 0.84^c^	29.31 ± 1.16^a^	25.80 ± 0.72^b^	24.99 ± 0.80^b^	21.26 ± 0.15^c^
Energy gain (Kcal)^9^	189.53 ± 7.90^b^	255.97 ± 5.23^a^	206.44 ± 5.36^b^	202.38 ± 11.30^b^	246.74 ± 3.68^a^
Energy utilization (%)^10^	20.85 ± 0.59^d^	33.91 ± 0.05^a^	27.50 ± 0.69^c^	25.55 ± 1.20^c^	30.63 ± 0.18^b^
Biometric parameters					
HSI (%)^11^	1.98 ± 0.12^c^	2.76 ± 0.15^b^	3.31 ± 0.34^a^	2.67 ± 0.26^b^	3.20 ± 0.18^a^
VSI (%)^12^	9.14 ± 0.14^ab^	8.95 ± 0.20^ab^	8.25 ± 0.34^b^	9.14 ± 0.01^ab^	9.71 ± 0.56^a^
Inter peritoneal fat (IPF) (%/body)^13^	2.71 ± 0.12^ab^	2.47 ± 0.28^b^	1.12 ± 0.13^c^	2.34 ± 0.19^b^	3.39 ± 0.47^a^

^1^Gain, g/fish = (final body weight − initial body weight). ^2^ADG, g/fish/day = (final body weight − initial body weight)/number of experimental days. ^3^SGR, %/fish/day = 100 × (ln(final body weight) − ln(initial body weight))/days. ^4^Condition factor = final body weight (g) /final body length^3^ (cm^3^). ^5^FI (g/fish^−1^) = feed intake (g/fish/day) = feed consumption (g)/average biomass (g) × days. ^6^FCR = feed conversion ratio = g feed consumed/g weight gained. ^7^PER, g = 100 × (total weight gain (g)/protein intake (g)). ^8^PPV, % = 100 × (protein gain (g) /protein intake (g)). ^9^Energy gain, Kcal = (energy content in fish carcass at the end) − (energy content in fish carcass at the start). ^10^Energy utilization, % = 100 − energy gain (kcal)∕energy intake (kcal). ^11^HSI, % = 100 × (liver weight (g)/fish body weight (g)). ^12^VSI, % = 100 × (viscera weight (g)/fish body weight (g)). ^13^Inter peritoneal fat (IPF), %/body = 100 × (intraperitoneal fat weight (g)/fish body weight (g)). ^*∗*^Values are means ± SEM; *N* = 3 readings per treatment. Values in the same row with a different superscript alphabets are significantly different (*P* < 0.05). Treatments = control diet CD (0 g WC or AC kg^−1^ feed), WC-1 (10 g WC kg^−1^ feed), WC-2 (20 g WC kg^−1^ feed), AC-1 (10 g AC kg^−1^ feed), and AC-2 (20 g AC kg^−1^ feed).

**Table 4:** The whole-body composition (dry matter, protein, lipid, and ash) of European seabass fed an experimental diet supplemented with different levels of wood charcoal (WC) and activated charcoal (AC), for 120 days.

Carcass composition parameter	Initial	Control (CD)	Wood charcoal		Activated charcoal	
			(WC-1)	(WC-2)	(AC-1)	(AC-2)
Moisture (%)	61.58 ± 0.17	65.50 ± 0.36^b^	64.47 ± 0.33^c^	65.40 ± 0.18^b^	66.06 ± 0.72^a^	62.04 ± 0.25^d^
Dry matter (%)	38.42 ± 0.35	34.50 ± 0.36^bc^	35.53 ± 0.33^b^	34.61 ± 0.18^bc^	33.94 ± 0.72^c^	37.96 ± 0.25^a^
Protein (%)	22.89 ± 0.35	18.82 ± 1.26^b^	18.82 ± 0.72^b^	19.22 ± 0.85^a^	19.23 ± 0.28^a^	18.78 ± 0.19^b^
Ether extract (%)	9.01 ± 0.18	10.61 ± 1.72^c^	12.46 ± 1.13^b^	11.08 ± 1.36^bc^	10.45 ± 0.62^c^	14.87 ± 0.52^a^
Ash (%)	5.44 ± 0.22	4.31 ± 0.17^a^	3.64 ± 0.17^b^	3.71 ± 0.34^b^	3.55 ± 0.33^b^	3.65 ± 0.15^b^
Carcass energy (Kcal/100 g)^1^	558.7 ± 7.4	600.70 ± 9.18^c^	629.86 ± 6.70^ab^	615.43 ± 8.18^bc^	610.22 ± 4.43^bc^	648.92 ± 4.62^a^

^1^Carcass energy (kcal∕100 g DM) = (protein% × 5.64) + (ether extract% × 9.44).  ^*∗*^Values are means ± SEM, *n* = 3 readings per treatment. Values in the same row with a different superscript alphabets are significantly different (*P* < 0.05). Treatments = control diet CD (0 g WC or AC kg^−1^ feed), WC-1 (10 g WC kg^−1^ feed), WC-2 (20 g WC kg^−1^ feed), AC-1 (10 g AC kg^−1^ feed), and AC-2 (20 g AC kg^−1^ feed).

**Table 5:** The effects of dietary commercial wood charcoal (WC) and activated charcoal (AC) on the liver serum enzymes and kidney function of European seabass fed an experimental diet supplemented with different levels WC and AC, for 120 days.

Parameters	Control (CD)	Wood charcoal		Activated charcoal	
		(WC-1)	(WC-2)	(AC-1)	(AC-2)
Liver serum enzymes					
ALP (U/L)^1^	84.5 ± 0.29^a^	66.0 ± 2.31^c^	64.0 ± 1.15^c^	78.0 ± 1.15^b^	87.0 ± 3.46^a^
ALT (U/L)^2^	49.5 ± 0.29^a^	35.5 ± 0.87^b^	24.5 ± 2.02^c^	31.5 ± 0.87^b^	35.5 ± 3.75^b^
AST (U/L)^3^	78.5 ± 2.02^a^	65.5 ± 3.18^b^	49.0 ± 0.58^c^	57.5 ± 3.75^bc^	53.0 ± 3.46^c^
Indicators of kidney function					
Urea nitrogen (mmol/L)	21.0 ± 1.15^a^	18.0 ± 1.150^b^	15.5 ± 0.29^bc^	15.5 ± 0.87^bc^	13.0 ± 0.0^c^
Uric acid (mg/dL)	3.41 ± 0.11^a^	3.04 ± 0.02^b^	2.20 ± 0.06^c^	3.14 ± 0.10^b^	2.38 ± 0.06^c^
Ammonia (mmol/L)	34.50 ± 2.60^a^	21.50 ± 1.44^b^	19.95 ± 1.41^b^	18.00 ± 1.73^b^	17.84 ± 2.21^b^

^1^ALP (U/L) = alkaline phosphatase. ^2^ALT (U/L) = glutamic–pyruvic transaminase. ^3^AST (U/L) = aspartate aminotransferase.  ^*∗*^Values are means ± SEM, *N* = 3 readings per treatment. Values in the same row with a different superscript alphabets are significantly different (*P* < 0.05). Treatments = control diet CD (0 g WC or AC kg^−1^ feed), WC-1 (10 g WC kg^−1^ feed), WC-2 (20 g WC kg^−1^ feed), AC-1 (10 g AC kg^−1^ feed), and AC-2 (20 g AC kg^−1^ feed).

**Table 6:** Antioxidative stress activity and digestive enzyme parameters of European seabass fed experimental diet supplemented with different levels of wood charcoal (WC) and activated charcoal (AC), for 120 days.

Antioxidative stress indicators	Control (CD)	Wood charcoal		Activated charcoal	
		(WC-1)	(WC-2)	(AC-1)	(AC-2)
Antioxidative stress					
TAC (mmol/L)^1^	3.41 ± 0.04^a^	2.19 ± 0.17^c^	2.85 ± 0.04^b^	2.77 ± 0.10^b^	2.18 ± 0.04^c^
MAD (nmol/mL)^2^	4.17 ± 0.03^d^	4.65 ± 0.07^c^	5.62 ± 0.20^b^	5.83 ± 0.15^b^	6.08 ± 0.05^a^
SOD (U/mL)^3^	1.47 ± 0.09^a^	1.20 ± 0.24^b^	1.06 ± 0.04^c^	1.01 ± 0.08^c^	1.24 ± 0.10^b^
CAT (U/mL)^4^	476.7 ± 3.53^a^	449.3 ± 6.17^b^	455.0 ± 22.34^b^	456.0 ± 10.4^b^	433.3 ± 25.2^c^
GPx (U/mL)^5^	2.45 ± 0.21^c^	2.70 ± 0.11^b^	3.38 ± 0.14^a^	2.77 ± 0.22^b^	3.41 ± 0.04^a^
Digestive enzyme					
Lipase (U/mL)	20.33 ± 1.20^c^	25.33 ± 1.45^a^	22.00 ± 1.15^b^	22.67 ± 1.20^b^	21.67 ± 0.88^bc^
Amylase (U/mL)	41.33 ± 1.20^d^	52.00 ± 2.08^a^	47.87 ± 1.73^c^	49.33 ± 1.20^b^	48.00 ± 2.33^c^

^1^ TAC = total antioxidative capacity. ^2^MAD = malondialdehyde. ^3^SOD = superoxide dismutase. ^4^CAT = catalase. ^5^GPx = activity of glutathione peroxidase.  ^*∗*^Values are means ± SEM, *N* = 3 readings per treatment. Values in the same row with a different superscript alphabets are significantly different (*P* < 0.05). Treatments = control diet CD (0 g WC or AC kg^−1^ feed), WC-1 (10 g WC kg^−1^ feed), WC-2 (20 g WC kg^−1^ feed), AC-1 (10 g AC kg^−1^ feed), and AC-2 (20 g AC kg^−1^ feed).

**Table 7:** Effects of dietary commercial wood charcoal (WC) and activated charcoal (AC) on serum immune and hematological parameters of European seabass fed an experimental diet supplemented with different levels of WC and AC, for 120 days.

Serum biochemical parameter	Control (CD)	Wood charcoal		Activated charcoal	
		(WC-1)	(WC-2)	(AC-1)	(AC-2)
Hb (mg/dL)	9.11 ± 0.05^a^	6.79 ± 0.18^c^	6.74 ± 0.05^c^	8.02 ± 0.03^b^	8.13 ± 0.05^b^
Total protein (mg/dL)	135.0 ± 3.46^c^	171.0 ± 6.35^b^	165.5 ± 2.60^b^	177.5 ± 2.02^ab^	185.0 ± 4.04^a^
Albumin (mg/dL)	1.425 ± 0.06^b^	1.655 ± 0.03 ^a^	1.455 ± 0.03^b^	1.560 ± 0.06^ab^	1.670 ± 0.08^a^
Glucose (mg/L)	113.1 ± 2.02	110.9 ± 2.60	111.4 ± 3.75	111.0 ± 3.58	112.7 ± 3.46
Globulin (mg/dL)	1.65 ± 0.09^c^	2.74 ± 0.06^a^	2.80 ± 0.13^a^	2.93 ± 0.03^a^	2.56 ± 0.65^b^
Complement 3 (mg/dL)	7.35 ± 0.58^c^	12.40 ± 0.52^a^	8.50 ± 0.40^b^	8.90 ± 0.55^b^	11.75 ± 0.55^a^
Calcium (mg/dL)	13.35 ± 0.49^a^	13.19 ± 0.28^a^	12.01 ± 0.00^c^	12.56 ± 0.05^b^	11.62 ± 0.26^d^

^*∗*^Values are means ± SEM, *N* = 3 readings per treatment. Values in the same row with a different superscript alphabets are significantly different (*P* < 0.05). Treatments = control diet CD (0 g WC or AC kg^−1^ feed), WC-1 (10 g WC kg^−1^ feed), WC-2 (20 g WC kg^−1^ feed), AC-1 (10 g AC kg^−1^ feed), and AC-2 (20 g AC kg^−1^ feed).

## Data Availability

The data supporting the findings of this study are available from the corresponding author and may be provided upon reasonable request.
